# Clustering of smoking, alcohol consumption and weight gain in pregnancy: prevalence, care preferences and associated factors

**DOI:** 10.1186/s12884-023-06090-7

**Published:** 2023-11-17

**Authors:** Clare Desmet, Renee Reynolds, Jenna Hollis, Milly Licata, Justine Daly, Emma Doherty, Belinda Tully, Christophe Lecathelinais, John Wiggers, Melanie Kingsland

**Affiliations:** 1 Population Health, Hunter New England Local Health District, Locked Bag 10, Wallsend, NSW 2287 Australia; 2 Population Health Research Group, Hunter Medical Research Institute, New Lambton Heights, NSW 2305 Australia; 3 School of Medicine and Public Health, College of Health, Medicine and Wellbeing, The University of Newcastle, Callaghan, NSW 2308 Australia; 4 Armajun Aboriginal Health Service, Inverell, NSW 2360 Australia; 5 Gomeroi Nation, Inverell, Australia

**Keywords:** Smoking, Alcohol, Gestational weight gain, Australia, Care preference

## Abstract

**Background:**

Smoking, alcohol consumption and weight gain outside recommendations during pregnancy are preventable health risk factors associated with poorer health outcomes for mother and infant. Clustering of these risk factors further increases the risk and severity of outcomes. Limited research has explored the characteristics of pregnant women that are associated with clustering of these risks and women’s preferences for receiving support. This paper aimed to determine: (i) the prevalence of clustered preventable risk factors; (ii) associations between maternal characteristics and presence of clustered risk factors; and (iii) women’s preferences for receiving care for clustered risk factors.

**Methods:**

A cross-sectional survey was undertaken with women who had recently given birth in public maternity services in New South Wales, Australia. Descriptive statistics were used to assess prevalence of clustered risk factors and care preferences. Associations between the presence of clustered risk factors and maternal characteristics were assessed using multiple regression analyses.

**Results:**

Of the 514 women who completed the survey, 52% reported one preventable health risk factor and 10% and 2% reported two or three. For women with two or more risk factors, the most common combination was alcohol consumption and gestational weight gain outside of recommendations (50%, n = 30). One characteristic had an association with the presence of clustered risk factors. Most women (77%, n = 46) with clustered risk factors indicated they wanted support for these health risks. Preferences for support addressing some or all risk factors, and whether the support was sequential or simultaneous, were not associated with particular risk factor combinations.

**Conclusions:**

Around one in eight women reported clustered preventable risk factors during pregnancy, most of whom would like support to address these risks. There was only one association between maternal characteristics and clustered risk factors. This suggests a need for antenatal care that is women-centred and caters for a diverse profile of clustered risks and varied preferences for care.

## Background

Smoking, alcohol consumption and gaining weight outside of recommendations during pregnancy are all preventable health risk factors that collectively relate to SNAP health behaviours (i.e., smoking, nutrition, alcohol and physical activity; considering that nutrition and physical activity are two modifiable behaviours that influence gestational weight gain) [1]. Studies, and systematic review have shown they are associated with adverse pregnancy and health outcomes for both the mother and child including pre-term labour [1–3], low birth weight [2, 3], stillbirth [4, 5], miscarriage [6] and macrosomia [7]. The global prevalence of smoking during pregnancy is estimated to be 1.7%, however this differs significantly by country, ranging from 0.2% in Tanzania, to 38.4% in Ireland [8]. It is estimated that 9.8% of pregnant women consume alcohol world-wide, with country level consumption ranging from 0% in Oman, United Arab Emirates, Saudi Arabia, Qatar and Kuwait, to 60% in Ireland [9]. Globally, up to 70% of pregnant women gain gestational weight outside of the Institute of Medicine (IOM) Guidelines [10], with 72%, 69% and 68% of pregnant women in the United States of America, Europe and Asia respectively, gaining weight outside of IOM recommendations [11]. In Australia, the prevalence of maternal smoking is 10% [12], alcohol consumption after pregnancy recognition is 15% [13] and gestational weight gain outside of recommendations is > 60% [10, 14, 15].

In addition to the prevalence of individual preventable health risk factors during pregnancy, a number of studies have shown that 23–56% of pregnant women report clustering of risk factors including smoking tobacco, cannabis, alcohol, pregnancy weight gain outside of recommendations, psychoactive drugs and caffeine [3, 16, 17]. As the number of such risk factors increases, so does the odds and severity of poor obstetric and neonatal outcomes [3]. For instance, in a 2016 retrospective cohort study involving 6822 women in New Zealand, women with more than one risk factor (body mass index (BMI) and weight change outside recommendations, smoking, alcohol consumption or physical activity below recommendations) had higher odds of: caesarean birth (OR = 2.03 for two risk factors and OR = 2.86 for three risk factors), pre-term birth (OR = 2.87 with two or three risk factors), a small for gestational age infant (OR = 2.39 for two risk factors and OR = 6.24 for three risk factors), and a low birth weight infant (OR = 2.24 for two risk factors and OR = 3.86 for three risk factors), compared to women with no risk factors [3].

Links between smoking and alcohol consumption among pregnant women have been shown in past studies by Passey et al. [16] and Lange et al. [8]. In an Australian cross-sectional study of 257 pregnant women who identified as Aboriginal or had an Aboriginal partner, Passey et al. found that women who smoked cigarettes had higher odds of consuming alcohol (OR 4.32; 95%CI 2.12, 9.13) compared to women who did not smoke [16]. Lange et al. undertook secondary analysis of a cross-sectional population based survey of 22,962 Canadian women, and found co-occurrence of alcohol use and smoking during pregnancy [18]. There is also recent evidence of clustering of smoking and gestational weight gain outside of recommendations [19]. A pilot study of 58 pregnant women in the USA reported that women who continued to smoke during pregnancy were statistically more likely to have inadequate gestational weight gain prior to delivery compared to women who had quit smoking during pregnancy (p = 0.004) [19]. No studies to date have reported on the clustering of smoking, alcohol consumption and gestational weight gain as a group of preventive health risks. The only studies that have explored these risks, have done so in the context of additional risks including cannabis [16], caffeine and psychoactive drugs [17]. Given the increasing risk of poor outcomes with clustering of smoking, alcohol and gestational weight gain outside of recommendations in pregnancy, an understanding of the prevalence of individual and clustered risk is needed to quantify the need for preventive health care intervention as part of routine antenatal care.

It is also largely unknown whether particular groups of pregnant women experience clustering of preventable health risk factors more than others. A limited number of studies have examined the association between any clustered risk factors and maternal characteristics. In a Canadian survey of 605 pregnant women, women with lower education and income levels, Indigenous or Metis background, and those who had previously given birth were more likely to report two or more drug related risk factors (alcohol, smoking, psychoactive drugs and/or caffeine) [17] during pregnancy. One Australian study of 257 pregnant women who identified as Aboriginal or had an Aboriginal partner, found that lower education level (less than year 10 at high school) and earlier initiation of substance use (less than 15 years old) were associated with a higher likelihood of using multiple substances (tobacco, alcohol, and/or cannabis) during pregnancy [16]. However neither of these studies reported any characteristics associated with the clustering of alcohol consumption and smoking specifically. Additionally Lange et al. in their analysis of the cross-sectional survey of 22,962 Canadian women found that having an extreme perceived amount of life stress was predictive of the co-occurrence of alcohol use and smoking during pregnancy (RRR = 4.40 (95% CI: 1.72–11.30, P = 0.0021)). They found no other associations with maternal characteristics (age, ethnicity, education, employment status, annual household income or marital status) [18]. No studies have examined any maternal characteristics associated with clustering of smoking, alcohol consumption and weight gain outside of recommendations during pregnancy. This information could assist in determining whether specific sub-groups of women require tailored antenatal care for these preventable health risks.

There is strong evidence that interventions delivered as part of antenatal care can reduce the prevalence of these risks [1, 20–22]. As such, the World Health Organisation’s recommendations for antenatal care advise that proactively addressing smoking, alcohol and weight before conception and in pregnancy is one of the most important ways healthcare providers can optimise outcomes for women and babies [23]. These recommendations provide evidence-based guidance for addressing smoking, alcohol consumption and gestational weight gain as singular risk factors. This single-risk factor approach is also reflected in national guidelines such as the United Kingdom’s National Institute for Health and Care Excellence (NICE) guidelines [24] and the Australian Clinical Practice Guidelines for antenatal care [25]. None of these guidelines provide guidance on how to address clustered risk factors [25]. It has been hypothesised that a concurrent approach to providing care for clustered risks may be more efficient in terms of clinical capacity and time, and maximise benefits achieved from antenatal care opportunities [26]. It has also been thought that such an approach may work synergistically to improve health behaviours and reduce disease burden and medical costs [27]. However, there is a paucity of evidence regarding women’s preference for care for clustered risk factors in terms of whether they would prefer to address risk factors separately or together [28]. Such an understanding would help inform the development and delivery of antenatal support that is most acceptable and therefore potentially more effective in modifying these risk factors in pregnancy.

This study aimed to examine:

- Prevalence and clustering of three preventable health risk factors (smoking, alcohol consumption and gestational weight gain outside of recommendations) in pregnant women attending public antenatal clinics.
- Associations between maternal characteristics and presence of clustered risk factors.
- Women’s preferences for antenatal care addressing clustered risk factors.

Note, that from this point forward, Aboriginal and Torres Strait Islander women and infants are referred to as ‘Aboriginal’, however we acknowledge their individual cultural identities.

## Methods

### Design and setting

A cross sectional survey was undertaken between September 2018 and February 2019 with women who had recently given birth at public maternity services in urban and rural communities within the Hunter New England Local Health District of New South Wales (NSW), Australia.

### Ethical approval

The study was approved by the Hunter New England Human Research Ethics Committee (16/11/16.407), the Aboriginal Health and Medical Research Council (1236/16), and the University of Newcastle Human Research Ethics Committee (H-2017-0032).

### Participants and recruitment

Women who had previously participated in a survey on the antenatal care they received during pregnancy [29] and consented to further contact from the research team were invited to participate in the study. Women were eligible to participate if they: were at least 18 years of age, attended a public maternity service for antenatal care and had given birth between 8 and 21 weeks prior. Due to ethical considerations regarding participant burden, women were also excluded if they had experienced an adverse pregnancy related outcome, (defined as stillbirth or miscarriage). Each week during the study period, an information statement was posted to eligible women, outlining the purpose of the study and inviting them to participate. A toll free number was included in the information statement allowing women to call and decline participation in the survey. One week after the information statement was mailed, women were contacted via telephone and invited to participate in a telephone survey.

Attempts to contact women were conducted over a two week period with up to 10 contact attempts made. Women could decline participation at any point.

### Data collection procedure and measures

Surveys were conducted by trained interviewers and Aboriginal women were offered the option of a female Aboriginal interviewer. Survey questions were based on those in Australian National and state surveys [30, 31], used validated tools where available and were piloted prior to use. The survey was reviewed for cultural appropriateness for Aboriginal and Torres Strait Islander women.

Women reported their smoking risk status and were classified as ‘smoking during pregnancy’ if they reported smoking ‘at the time they found out they were pregnant’, ‘at their first appointment’ and/or ‘at the time their baby was born’. Women were classified as ‘consuming alcohol during pregnancy’ if they answered ‘Monthly or less’, ‘2–4 times a month’, ‘2–3 times a week’ or ‘4 or more times a week’ to the question ‘How often did you have a drink containing alcohol after you knew you were pregnant?’ [32]. Women reported their height, pre-pregnancy weight and weight at the time of birth, singleton/multiple pregnancy, and weeks’ gestation at time of birth. Women with more than one preventable health risk factor during pregnancy were asked ‘Would you like to have received care for these health behaviours one at a time or all at the same time?’ If they responded with ‘one at a time’, they were asked ‘In which order would you like to have received support for these health behaviours?’. The definitions used in this study for consuming alcohol and smoking during pregnancy follow the Australian Clinical Practice Guidelines for Pregnancy Care [25] that state that that not drinking alcohol, and quitting smoking completely, have the greatest health benefits. Similarly, the Australian Guidelines to Reduce Health Risks from Drinking Alcohol [33] advise that ‘if you are pregnant or planning a pregnancy, you should not drink any alcohol in order to prevent the risk of damage to the developing baby’. The Australian Government Department of Health and Aged Care specify ‘there is no safe amount of smoking’ [34]. Therefore, these thresholds have been used to estimate the proportion of pregnant women with clustering of these risk factors.

Educational status and information on Aboriginal status of mother and infant were collected in the previous survey women participated in during their pregnancy [35]. Clinical records were accessed to collect the woman’s postcode, date of birth and model of antenatal care.

### Statistical analysis

Data analysis was conducted using SAS 9.3 [36]. Residential postcode was used to classify geographical remoteness using the Australian Statistical Geography Standard [37] and socio-economic status was classified using the Socio-economic Indexes for Area (SEIFA - the Index of Relative Socio-economic Advantage and Disadvantage (IRSAD)) (most vs. least disadvantaged by dichotomising on the NSW median) [38]. Condensed response categories were created for Aboriginal origin of women and infants (‘Aboriginal or Torres Strait Islander origin or both’ or ‘non-Indigenous’) and education level (‘high school or less’ or ‘tech certificate or diploma’ or ‘university or college degree or higher’). Antenatal model of care was used to indicate women’s pregnancy risk level with midwifery-led care classified as ‘low risk pregnancy’ and medical led-care classified as ‘high risk pregnancy’. Women allocated to Aboriginal Maternal and Infant Health Services were excluded from the pregnancy risk variable as such services see women with low and high risk pregnancies. Pre-pregnancy weight and height were used to calculate a woman’s pre-pregnancy BMI (underweight, healthy weight, overweight and obesity) [39]. Total gestational weight gain was calculated as the difference between self-reported weight pre-pregnancy and weight at the time of birth. Women were classified as gaining weight within or below/above their recommended gestational weight gain according to the IOM guideline ranges [39] for singleton pregnancies, based on pre-pregnancy BMI and adjusted for weeks’ gestation. IOM guidelines for weight gain are (gain of 12.5–18 kg for underweight women [BMI < 18.5]; 11.5–16 kg for normal-weight women [BMI 18.5–24.9]; 7–11 kg for overweight women [BMI 25-29.9]; and 5–9 kg for obese women [BMI ≥ 30]). Clustered risk factors were defined as having two or more risk factors.

Descriptive statistics were used to report prevalence and clustering of risk factors and care preferences.

A multivariable logistic regression model was used to identify associations between maternal characteristics and whether women had clustered risk factors (i.e. 0–1 reported risk factors vs. 2–3 risk factors). The independent variables included in the model are: age, pre-pregnancy BMI, Aboriginal status of the mother, Aboriginal status of the infant, education level index of disadvantage, first pregnancy, antenatal model of care, and woman’s geographic remoteness. Associations between each characteristic and number of risks (0,1,2,3 separately) were also explored using Fisher’s Exact Tests. An alpha level of 0.05 was set to denote statistical significance with no further adjustment made to the alpha level for multiple testing due to the exploratory nature of the study. Fisher’s Exact Test was used to detect any significant differences between care preferences of women with different combinations of multiple risk factors.

## Results

### Participants

Over the six month survey period, 973 women were sampled, of which 688 (71%) were able to be contacted. Of the 686 women deemed eligible upon contact (99.7%), 514 (75%) consented to and completed the survey.

The younger the woman, the less likely they were to participate (OR: 0.96 95% CI 0.93–0.98) (p < 0.001). Level of education was also associated with participation (p < 0.001) with those completing high school or less (OR: 2.60 95%CI 1.88–3.61), or a Tech certificate or diploma (OR: 1.80 95%CI 1.33–2.44), having greater odds of not participating compared to those with a University level education.

### Prevalence and clustering of preventable health risk factors

**Fig. 1:**
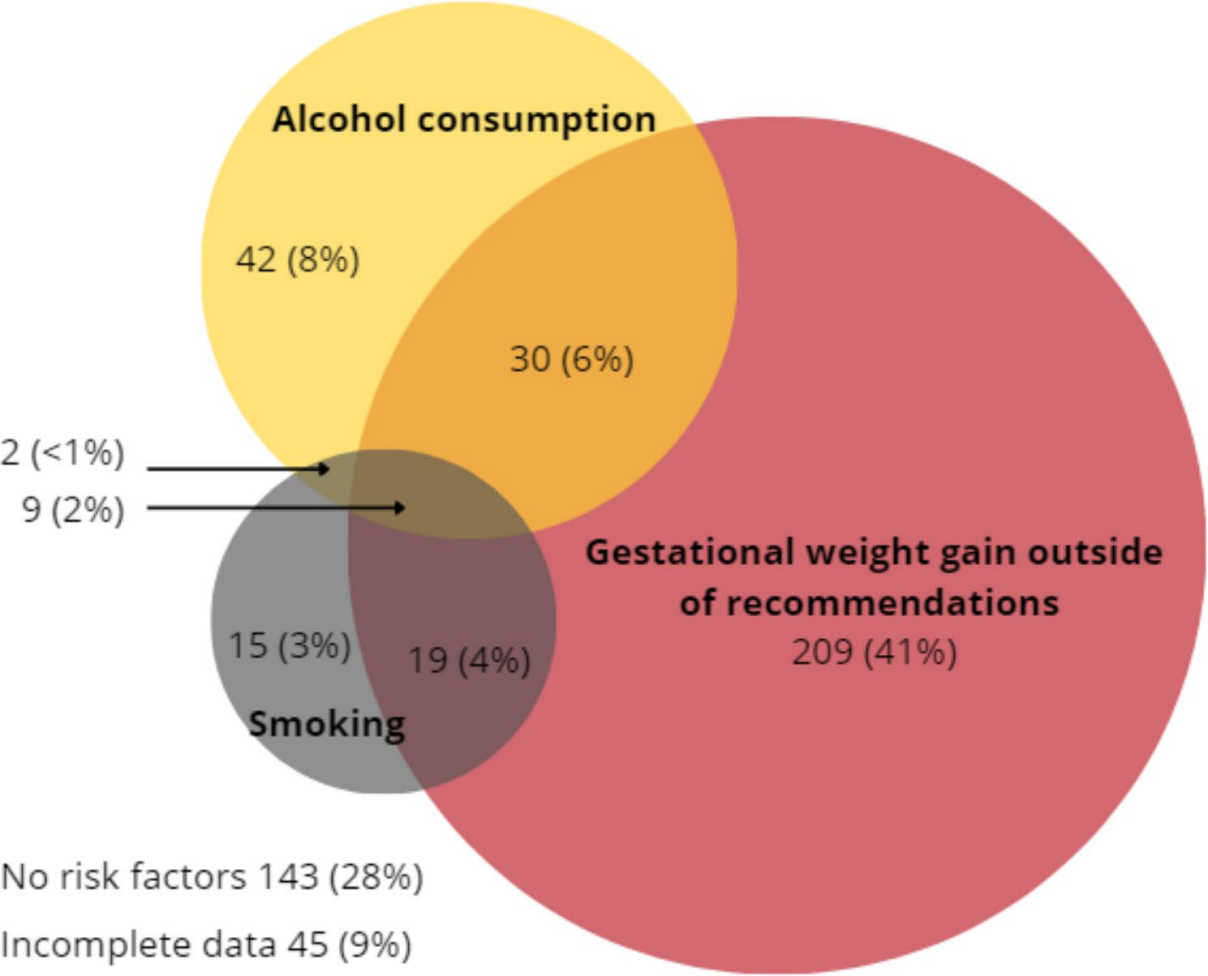
Self-reported risk factors during pregnancy

Of all participants, 8.8% (45/514) reported smoking at any time during pregnancy, 16.1% (83/514) reported consuming alcohol during pregnancy and 57.1% (267/469) reported gestational weight gain that was above or below recommended levels. Forty-five participants had incomplete data for weight and could not have gestational weight gain calculated. Sixty women (11.6%) reported two or more preventable health risk factors. As shown in Fig. 1, the proportion of women reporting two risk factors were: <1% (n = 2) for smoking and alcohol consumption, 4% (n = 19) for smoking and weight gain outside of recommendations, and 6% (n = 30) for alcohol consumption and gestational weight gain outside recommendations. Nine women (2%) reported all three preventable health risk factors during pregnancy.

### Associations between maternal characteristics and clustering of preventable risk factors

As shown in Table 1, the results of the multiple regression found that women who were pregnant with an Aboriginal baby had greater odds of reporting two or more risk factors (OR 6.38, 95%CI 1.77–22.94, p = 0.005). There were no other significant associations between maternal characteristics and reporting of clustered risk factors.

**Table 1:** Associations of maternal characteristics with clustering of risk factors using multiple regression

Maternal characteristic	n (%)			2-3 Risk Factors OR (95% CI)	*p*
	Overall (N=514)	0-1 risk factors (N=454)	2-3 risk factors (N=60)		
***Age (mean (SD))***	30.42 (4.94)	30.38 (4.90)	30.73 (5.26)	1.03 [0.97–1.10]	0.33
***Pre-pregnancy BMI (mean (SD))*** †	26.22 (6.30)	26.24 (6.37)	26.06 (5.82)	0.98 [0.93-1.03]	0.36
***Aboriginal Status of mother***					0.96
Aboriginal	25 (4.86%)	22 (88.00%)	3 (12.00%)	0.24 [0.03–1.74]	
Non-Aboriginal	489 (95.15%)	432 (88.34%)	57 (11.66%)		
***Aboriginal status of infant***					0.005
Aboriginal	41 (7.99%)	33 (80.48%)	8 (19.51%)	6.38 [1.77-22.94]	
Non-Aboriginal	472 (92.01%)	420 (88.98%)	52 (11.02%)		
***Education level***					0.16
High school or less	116 (22.57%)	101 (87.07%)	15 (12.93%)	1.80 [0.82-3.97]	
Tech certificate or diploma	177 (34.44%)	151 (85.31%)	26 (14.69%)	1.62 [0.81-3.23]	
University or college degree or higher	221 (43.00%)	202 (91.40%)	19 (8.60%)		
***Index of disadvantage***					0.97
Most disadvantaged	265 (51.56%)	230 (86.79%)	35 (13.21%)	1.01 [0.52-1.96]	
Least disadvantaged	249 (48.44%)	224 (89.96%)	25 (10.04%)		
***First pregnancy***					0.49
Yes	202 (39.30%)	182 (90.10%)	20 (9.90%)	0.80 [0.43-1.51]	
No	312 (60.70%)	272 (87.18%)	40 (12.82%)		
***Antenatal Model of Care***					0.43
Low risk (midwives)	340 (66.15%)	303 (89.12%)	37 (10.88%)	0.72 [0.34-1.53]	
High Risk (Medical clinic)	165 (32.10%)	142 (86.06%)	23 (13.94%)		
***Woman’s geographic remoteness***					0.29
Inner/Outer Regional/Remote	139 (27.04%)	117 (84.17%)	22 (15.83%)	1.65 [0.65-4.17]	
Major city	375 (72.96%)	337 (89.87%)	38 (10.13%)		

† Forty five missing responses

### Women’s preferences for antenatal care addressing clustered risk factors

**Fig. 2:**
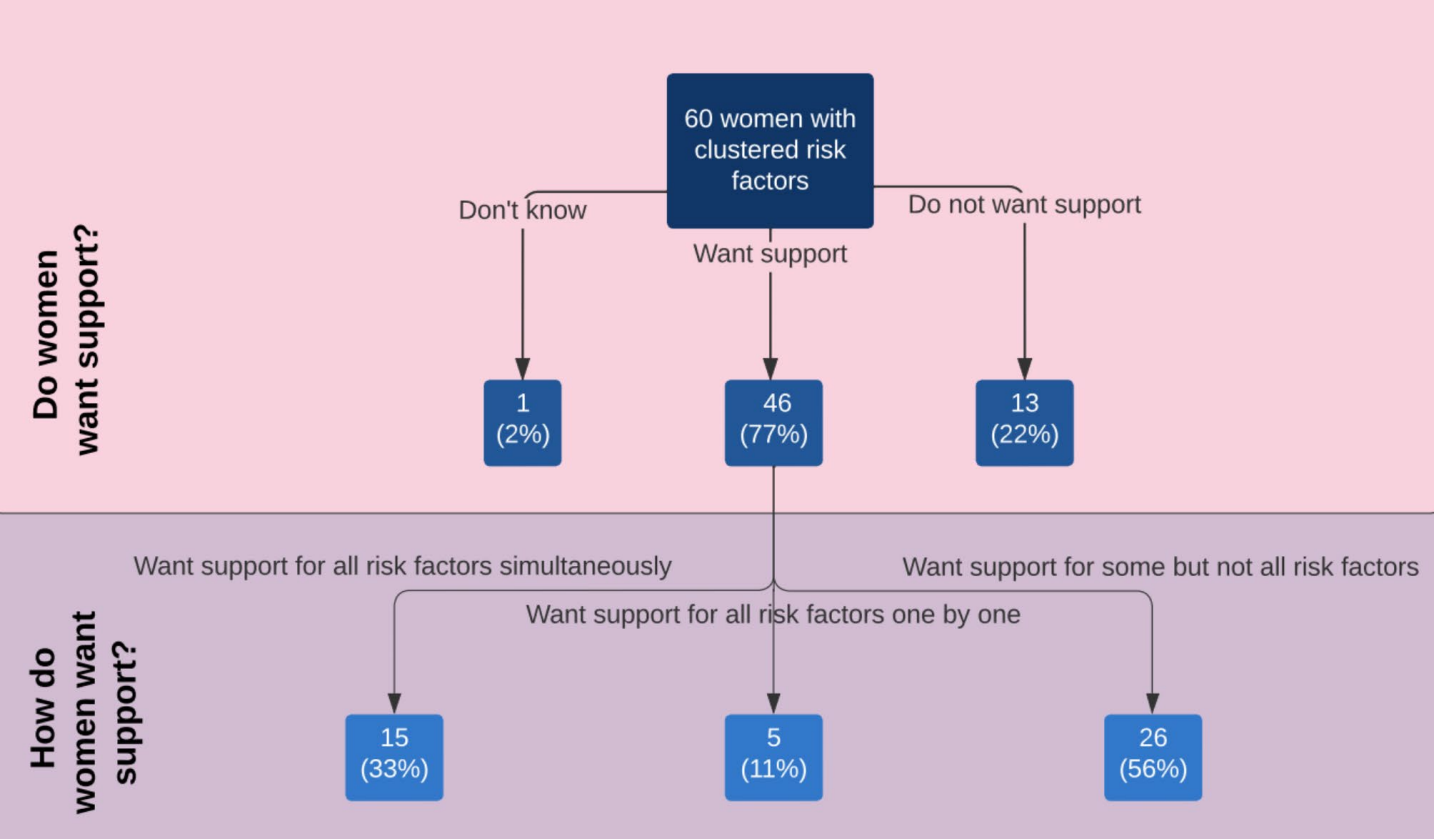
Care preferences of women with clustered preventable health risk factors in pregnancy

As shown in Fig. 2, of the 60 participants who reported two or more risk factors during their pregnancy, 46 (76.7%) indicated that they would have liked support to address at least one of these risk factors. Of those who wanted support, 44% wanted support for all risk factors and 56% for at least one but not all risk factors. Of those who wanted support for all risk factors, three quarters (75%) wanted support for all risk factors at the same time and one quarter (25%) wanted support for one risk at a time. There were no significant differences found between care preferences and combination of risk factors (p = 0.33).

## Discussion

This is the first study to examine the clustering of the preventable risk factors of smoking, alcohol consumption and weight gain outside of recommendations during pregnancy, and women’s preferences for addressing these in routine antenatal care. Around one in eight women reported two or more preventable risk factors during pregnancy with the most common cluster being gestational weight gain outside of recommendations and alcohol consumption. Multiple regression showed women who were pregnant with an Aboriginal baby had greater odds of reporting two or more risk factors, when compared to women pregnant with non-Aboriginal babies (OR 6.38, 95%CI 1.77–22.94, p = 0.005). However, given the small numbers of women having Aboriginal babies (n = 41) and lack of data on other contextual factors, more work needs to be done to understand these results and their importance. Most women with clustered risk factors indicated that they wanted support to address their risk factors, with nearly half wanting support for all risk factors. Preferences for support were not associated with particular risk factor combinations.

The prevalence of single risk factors during pregnancy reported in this study are comparable to previous Australian studies (smoking: 9% compared to 10% [12]; alcohol consumption after pregnancy recognition: 15% compared to 15% [13]; gestational weight gain outside of recommendations: 57% compared to 64% [14]). One in eight women (11.6%, n = 60) reported clustering of two or more of these preventable risk factors, with 2% (n = 9) of women reporting all three preventable risk factors. Alcohol consumption and gestational weight gain outside recommendations was reported as the most prevalent cluster (6%, n = 30). No previous studies have reported on clustering of these three preventable risk factors, so there is currently limited ability to compare these findings as a whole to those of other cohorts of pregnant women. The two studies which allow the closest comparison of multiple risk factors during pregnancy stated that 44% of pregnant Aboriginal women reported using two or three substances (tobacco, alcohol or cannabis) [16] and that 36%, 16% and 4% of women reporting using 2, 3 or 4 substances (caffeine, alcohol, tobacco and psychoactive drugs) [17]. Although the prevalence of multiple risk factors in each of these studies is higher than the 11.6% found in this study, the lack of consistent risk factors examined, precludes any further comparisons. Given the limited available evidence, more research is warranted to further confirm these findings and to further explore the relationship between these preventable health risk factors during pregnancy.

This study found that there were very limited associations found between characteristics of women and the presence of clustered risk factors in this study. While previous research has indicated some associations between maternal characteristics and individual risk factors such as years of schooling and age of initiating tobacco [16], and education, income, Aboriginal or Metis background, and those not living with a partner [17], it is difficult to directly compare studies due to differences in risk factors examined and populations included. The small number of women with clustered risk factors in this study also reduced the power to confirm any associations. Further studies, with larger sample sizes are required to identify any particular groups that may have increased prevalence of clustered risks and may need additional support. Until further knowledge is gained, a routine and universal approach to assessment and offer of support to all women is warranted.

Most participants (77%) with clustered, preventable risk factors reported that they would like support to address these risk factors. However, care preferences for addressing multiple risk factors were varied, with 33% of participants with clustered risks wanting to address all at once, 11% wanting to address all but only one at a time and 56% wanting to only address one/some of their risk factors. These findings of varied preferences may be best considered in the context of women-centred care, which is a philosophy that underpins midwifery practice. Whilst the interpretation of women-centred care varies between countries, the concept is generally accepted globally as evidenced by studies from Australia, Ireland, Japan, Netherlands, New Zealand, South Africa, Sweden, Switzerland, the UK and the USA [40]. In Australia, the Council Of Australian Governments (COAG) Health Council (Department of Health) states that woman centred care is informed by three areas in shared decision making (1) a woman’s preference, (2) evidence as it applies to the woman, and (3) context of care provision [41]. By considering preference for support to address clustered preventative risk factors, antenatal staff are following the COAG principle specifying that “women’s choices and preferences are sought and respected throughout maternity care” [41].

While women centred care emphasises the importance of women’s preferences, there remains a lack of evidence for applying this approach to care addressing multiple risk factors during pregnancy, with inconsistent and limited evidence as to whether simultaneous or sequential approaches are most effective in reducing the prevalence of risk factors [42]. Previous reviews suggest that addressing one behaviour where motivation is high, could positively influence the modification of other behaviours [27, 43]. In studies of nutrition, physical activity and smoking behaviours of people in the USA and Germany, people who were in a later stage of the Transtheoretical Model (i.e. closer to actioning the change) for one behaviour, were more likely to be in a later stage for another behaviour as well [43]. Studies into addictive behaviours have also found that being treated for two addictions, resulted in greater long term sobriety (related to alcohol and other drugs) [27]. A systematic review examining multiple health behaviour change interventions clustered around adoption/cessation behaviours and lifestyle/addictive behaviours (smoking, diet, physical activity and alcohol consumption) concluded that there was limited evidence for the superiority of either simultaneous or sequential interventions, and proposed that both should be considered equally efficacious compared to usual/minimal care [42]. However, none of the trials included in these reviews have focussed on pregnant women. Two studies have examined support provided to pregnant women for multiple risk factors in Australia using a simultaneous approach. A 2010 quasi-experimental, two-group design study of 304 pregnant women found that women receiving an interactive, paper based resource were more likely to quit smoking, but had no differences in fruit and vegetable intake or physical activity compared to women who received usual care [44]. A 2012 randomised controlled trial of 360 pregnant women found that women who attended a workshop addressing dietary behaviours, gestational weight gain awareness, physical activity, smoking and breastfeeding were significantly more likely to report increased consumption of fruit and vegetables, a higher diet quality score and physical activity compared with women who received usual care [45]. Neither of these studies report on women’s preference for support for risk factors, nor stratify results by clustering of risk factors (i.e. women with two or more risk factors). They do however, suggest that a simultaneous approach may be effective in reducing behavioural risk factors in pregnant women. Further research is needed to determine whether a simultaneous or a sequential approach to care for pregnant women is effective in supporting behaviour change for more than one preventable risk factor. Further research is needed to determine whether a simultaneous or a sequential approach to care for pregnant women is effective in supporting behaviour change for more than one preventable risk factor.

The findings of this study and their generalisability should be considered in the context of a number of limitations. Women who were older and who had completed tertiary education were more likely to have participated compared to women who declined to participate. Women who identified as Aboriginal or Torres Strait Islander represented a lower proportion of participants in the study (4.86%) compared to the proportion in the total population of the Hunter New England region (7.1%) and this may have affected the study’s power when examining associations for this group of women and other subgroups [46]. Compared to Australian women giving birth in 2020 [15], women who completed the survey were of a similar age (30.42 years compared to 31.1 years), were similar in rates of living in a major city (72.96% compared to 73.1%) and represented a similar proportion of those having their first pregnancy (39.9% compared to 43.4%). Exclusion of women who had an adverse pregnancy outcome (including stillbirth and miscarriage) may have resulted in selection bias and underreporting of risks, given that stillbirth and miscarriage are associated with the risk factors examined in this paper [4, 6]. The study defined pregnancy risk according to the type and model of antenatal care that the woman received, which may not correspond with an individual’s actual medical risk. Nine percent (n = 45) of the sample did not have sufficient anthropometric data to calculate weight gain outside of recommendations and potential inaccuracy of self-reported height and weight may have influenced the correct classification of women who gained weight within and outside recommendations [47]. The small sample size of 60 women with clustered risk factors may have been insufficient to detect statistically significant differences when analysing data for care preferences. Women’s self-reported health risks may be influenced by social desirability bias which may have resulted in under-estimation of the prevalence of each risk factor. Potential recall bias may also have reduced these estimates, as this survey was completed 2–5 months post pregnancy. Future studies should be conducted throughout the pregnancy period to confirm these findings. Strengths include the random sample, high consent rate (74%) and use of validated and standardised measures. Further research with a larger, representative sample size of women with clustered risk factors is needed to confirm these findings. In addition, future studies with larger sample sizes should report on the prevalence of gestational weight gain below and above recommendations and examine risk factor clustering for these groups of women specifically.

## Conclusions

Around one in eight women reported multiple preventable health risk factors during pregnancy. There was only one association between maternal characteristics and clustered risk factors. Most women with clustered risk factors wanted support, but preferences for how this was provided varied, with some women preferring to address all risk factors simultaneously and others wanting to address them sequentially. There is a need for the health care system and antenatal care workers to be able to provide care for a diverse profile of clustered preventable health risks and preferences for care through women-centred approaches. Further evidence is needed to understand how to apply a concurrent approach to providing care for clustered risks where a woman chooses to address risks in such a way.

## Data Availability

The datasets used and/or analysed during the current study are available from the corresponding author on reasonable request.

## List of abbreviations

IOM: Institute of Medicine
BMI: Body Mass Index
NICE: National Institute for Health and Care Excellence
SEIFA: Socio-economic Indexes for Area
WCC: Woman Centred Care
COAG: Council Of Australian Governments
